# Establishment and inheritance of epigenetic transcriptional memory

**DOI:** 10.3389/fmolb.2022.977653

**Published:** 2022-09-02

**Authors:** Bethany Sump, Jason Brickner

**Affiliations:** Northwestern University, Evanston, IL, United States

**Keywords:** transcriptional memory, heritable histones, nuclear pore, *S. cerevisiae*, chromatin, chromosomes, epigentics

## Abstract

For certain inducible genes, the rate and molecular mechanism of transcriptional activation depends on the prior experiences of the cell. This phenomenon, called epigenetic transcriptional memory, accelerates reactivation and requires both changes in chromatin structure and recruitment of poised RNA Polymerase II (RNAPII) to the promoter. Forms of epigenetic transcriptional memory have been identified in *S. cerevisiae*, *D. melanogaster*, *C. elegans*, and mammals. A well-characterized model of memory is found in budding yeast where memory of inositol starvation involves a positive feedback loop between gene-and condition-specific transcription factors, which mediate an interaction with the nuclear pore complex and a characteristic histone modification: histone H3 lysine 4 dimethylation (H3K4me2). This histone modification permits recruitment of a memory-specific pre-initiation complex, poising RNAPII at the promoter. During memory, H3K4me2 is essential for recruitment of RNAPII and faster reactivation, but RNAPII is not required for H3K4me2. Unlike the RNAPII-dependent H3K4me2 associated with active transcription, RNAPII-independent H3K4me2 requires Nup100, SET3C, the Leo1 subunit of the Paf1 complex and can be inherited through multiple cell cycles upon disrupting the interaction with the Nuclear Pore Complex. The H3K4 methyltransferase (COMPASS) physically interacts with the potential reader (SET3C), suggesting a molecular mechanism for the spreading and re-incorporation of H3K4me2 following DNA replication. Thus, epigenetic transcriptional memory is a conserved adaptation that utilizes a heritable chromatin state, allowing cells and organisms to alter their gene expression programs in response to recent experiences over intermediate time scales.

## Introduction

Changes in gene expression programs that are epigenetic are defined to have a “stably heritable phenotype resulting from changes in a chromosome without alterations in the DNA sequence” (Nanney, 1958; Berger et al., 2009). Epigenetic regulation plays a role in almost all biological systems and is initiated by either changes in the environment or developmental signals. Signals can induce heritable changes in gene expression, which alter phenotypes, and these changes often require covalent modification of DNA or histones (Gold et al., 1963; Scarano et al., 1965; Edwards et al., 2017). The modifications most commonly required for epigenetic regulations are methylation of the DNA, post-translational modifications of histones, and non-coding RNAs (Bossdorf et al., 2007; Zhu and Reinberg, 2011; Edwards et al., 2017; Harvey et al., 2018).

The genome is functionally organized by epigenetic regulation. Epigenetic regulation distinguishes euchromatin, facultative heterochromatin, and constitutive chromatin. The stable gene expression states associated with differentiation, X-inactivation, imprinting, or stable boundaries between silent and expressed chromatin are all products of epigenetic regulation (Kellum and Schedl, 1991; Panning, 2008; Peters, 2014; Galupa and Heard, 2016; Atlasi and Stunnenberg, 2017). Transient environmental signals such as starvation or stress can induce heritable but impermanent alterations in gene expression (Ahmed et al., 2010; D’Urso et al., 2016; Sood and Brickner, 2017; Sump et al., 2022).

Epigenetic transcriptional memory is a phenomenon whereby cells inherit the ability for faster induction of certain genes if faced with a challenge that their ancestors overcame within the previous 4–14 cell divisions (Brickner et al., 2007; D’Urso et al., 2016; Gialitakis et al., 2010; Lämke and Bäurle, 2017; Light et al., 2013, 2010; Maxwell et al., 2014; Pascual-Garcia et al., 2017; Siwek et al., 2020; Sood and Brickner, 2017). While there are variations in memory systems, conserved mechanisms have been identified which include interactions with nuclear pore proteins, methylation of H3K4, and the poising of RNA Polymerase II at the promoter of the affected genes.

Epigenetic transcriptional memory in Saccharomyces cerevisiae occurs in response to nutrient signals such as starvation for inositol or amino acids (D’Urso et al., 2016; Light et al., 2010), a carbon source switch to galactose (Sood and Brickner, 2017), and stresses such as high salt (D’Urso et al., 2016; Gasch et al., 2000; Guan et al., 2012; McDaniel et al., 2018). In HeLa cells, cells exhibit memory in response to interferon gamma (Gialitakis et al., 2010; Light et al., 2013; Siwek et al., 2020). Memory in both yeast and human cells is associated with poised RNA Polymerase II and H3K4me2 and requires interaction with the nuclear pore protein Nup98 (yeast Nup100).

Additionally, in *Drosophila* S2 cells exhibit memory of exposure to the molting hormone ecdysone (Pascual-Garcia et al., 2022, 2017). A subset of the ecdysone-induced genes show greater transcriptional output upon a second exposure to the hormone, which is dependent on an interaction with the nuclear pore protein, Nup98. Active transcription during the initial treatment with ecdysone is not necessary for this effect, indicating that the benefit is not due to protein production but rather signaling or chromatin modifications in response to ecdysone (Pascual-Garcia et al., 2022).

Arabidopsis benefits from dehydration stress memory, allowing plants to better endure lack of water (Ding et al., 2013; Liu et al., 2014). Stress-response genes that exhibit dehydration stress memory maintain a high level of H3K4 methylation and a stalled RNA Polymerase II, which correlates with increased rates of transcription for those genes upon a second dehydration stress.

Lastly, in C. elegans, starvation induces the recruitment, but not initiation, of RNAPII over hundreds of genes, a phenomenon called “docking”. Docking is functionally similar to the recruitment of poised RNA Polymerase II observed in yeast and mammals during memory and has been suggested to maintain an open chromatin state over these promoters (Maxwell et al., 2014). Docked RNAPII is associated with development and growth genes that are rapidly upregulated when nutrients become available, promoting rapid recovery from starvation-induced L1 arrest (Maxwell et al., 2014).

### Establishment of *INO1* epigenetic transcriptional memory

**FIGURE 1:**
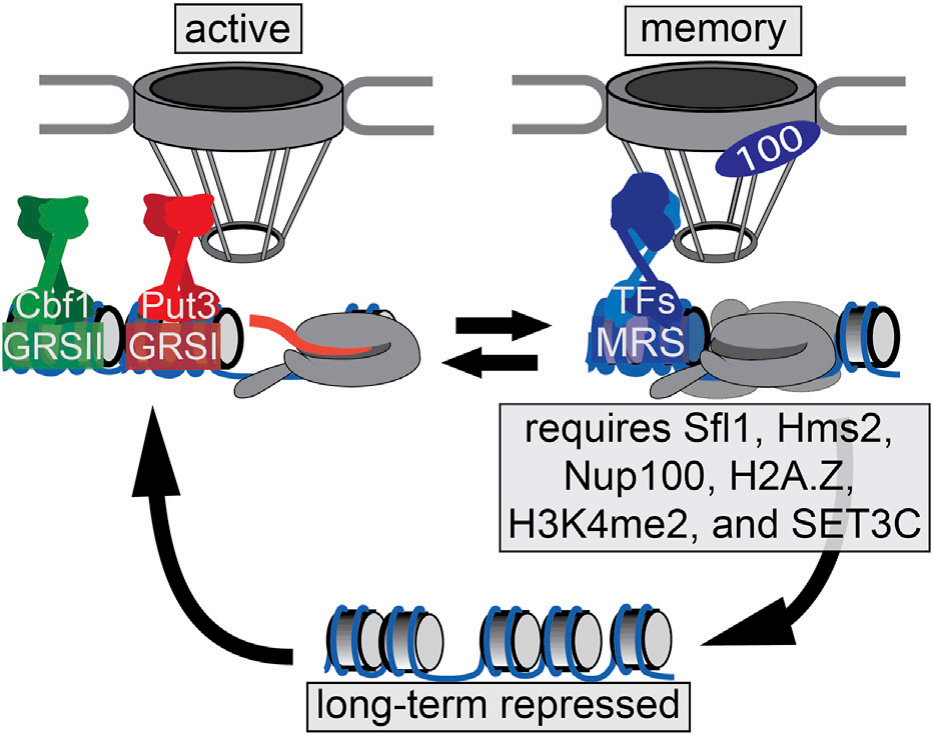
*INO1* transcriptional states. The *INO1* promoter region is depicted. When repressed, *INO1* is localized in the nucleoplasm and the chromatin is hypoacetylated and unmethylated. Upon activation, *INO1* is recruited the nuclear periphery through Put3- and Cbf1-dependent interaction with the NPC. The promoter and *INO1* gene are hyperacetylated and exhibit H3K4me1, H3K4me2, and H3K4me3. Upon repression, *INO1* remains associated with the NPC through an Sfl1/Hms2- and Nup100-dependent mechanism. This leads to incorporation of H2A.Z, deposition of H3K4me2 and the recruitment of poised RNAPII pre-initation complex (PIC), which allows more rapid re-activation. H3K4me2 also recruits SET3C, which is essential for memory.

One of the best-characterized models for epigenetic transcriptional memory is the gene *INO1* which codes for the protein, inositol-1 phosphate synthase, an essential protein in the production of inositol in budding yeast (Figure 1). In the presence of inositol, the *INO1*gene is repressed by Ume6 which binds to the Upstream Recruitment Sequence (URS), and also by Opi1, which interacts with the Ino2/Ino4 transcriptional activator bound to the UASINO (White et al., 1991; Jackson and Lopes, 1996; Graves and Henry, 2000). Repression is caused by recruitment of Sin3/Rpd3L (Lopes et al., 1993; Kadosh and Struhl, 1997; Rundlett et al., 1998; Heyken et al., 2005; Randise-Hinchliff et al., 2016). When the cell detects a lack of inositol, the *INO1* gene is relocated from the nucleoplasm to the nuclear periphery upon derepression. This is achieved by binding of two transcription factors, Put3 and Cbf1, to the Gene Recruitment Sequences, GRSI and GRSII respectively (Brickner and Walter, 2004; Ahmed et al., 2010; Randise-Hinchliff et al., 2016). These GRS elements act as DNA “zip codes”, cis-acting sequences that are both necessary and sufficient to confer positioning to the nuclear periphery. At the nuclear periphery, the *INO1* gene interacts with the Nuclear Pore Complex (NPC) in a Nup2-dependent manner (Brickner et al., 2019) and the transcriptional machinery is assembled, resulting in histone hyperacetylation, H3K4 methylation (i.e., H3K4me1, H3K4me2, and H3K4me3), and actively transcribing RNAPII.

Upon addition of inositol, instead of returning to a fully repressed state, the *INO1* gene adopts a memory state; the recently-repressed *INO1* gene remains localized at the nuclear periphery, interacting with the NPC. However, retention of the recently-repressed *INO1* gene at the nuclear periphery involves a different zip code (the Memory Recruitment Sequence, or MRS), bound to the transcription factors Sfl1 and Hms2, leading to interaction with the NPC dependent on the nuclear pore protein Nup100 (Light et al., 2010). The MRS, Nup100 and Sfl1/Hms2 are not required for transcription of *INO1*, but play a specific and essential role in memory (D’Urso et al., 2016; Light et al., 2010; Sump et al., 2022). While the active state of *INO1* is associated with histone acetylation and both tri- and dimethylation of H3K4, the memory state is associated with hypoacetylated histones, H3K4me2, as well as the incorporation of the histone variant H2A.Z upstream of the promoter (Brickner et al., 2007; D’Urso et al., 2016; Light et al., 2010). *INO1* memory is inherited through approximately four cell divisions. Cells with memory are able to more rapidly induce *INO1* (and other inositol-regulated genes) to overcome inositol starvation. This leads to a competitive fitness advantage over naïve cells during the adaptation to inositol starvation (Sump et al., 2022).

Methylation of histone H3 lysine 4 is catalyzed by histone methyltransferases related to Set1/COMPASS, a complex originally identified in budding yeast (Nislow et al., 1997; Briggs et al., 2001). Yeast COMPASS complex is made up of seven subunits (Set1, Bre2, Swd1, Swd2, Swd3, Sdc1, and Spp1). The Spp1 subunit is dispensable for H3K4 monomethylation and dimethylation, but is required for H4K4 trimethylation. Spp1 increases the ability of COMPASS to trimethylate lysine 4, perhaps by increasing the residency time of the complex (Soares et al., 2017). However, a remodeled form of COMPASS that lacks Spp1 (Spp1- COMPASS) is recruited to the *INO1* promoter during memory, whereas the complete COMPASS complex (Spp1+ COMPASS) associates with the *INO1* gene during activation.

The maintenance of H3K4me2 at the *INO1* promoter during memory is also dependent on the histone deacetylase complex, SET3C (D’Urso et al., 2016). There is no evidence that SET3C is responsible for removing the acetyl marks from the *INO1* promoter upon repression (D’Urso et al., 2016), but rather the evidence indicates that SET3C is acting as a “reader” of the H3K4me2 marks. The PHD domain of Set3 interacts with H3K4me2 (Kim and Buratowski, 2009). Conditional inactivation of Set3 causes a rapid loss of H3K4me2 from the *INO1* promoter during memory (D’Urso et al., 2016). Likewise, a mutation that disables the ability of the PHD domain to bind H3K4me2 disrupts *INO1* memory (D’Urso et al., 2016). Binding of SET3C to H3K4me2 may shield the marks from removal by the histone H3 lysine 4 demethylase, Jhd2 (Liang et al., 2007).

Importantly, the interactions between the Sfl1 transcription factor, Nup100, H3K4me2, and H2A.Z incorporation form a positive feedback loop, with all interactions being required for *INO1* memory (Sump et al., 2022). In this feedback loop, the interaction with the NPC is required for the H3K4 dimethylation and incorporation of H2A.Z at the *INO1* promoter (Sump et al., 2022). Additionally, H3K4me2 is required for the binding of Sfl1 during memory (Sump et al., 2022). Furthermore, H3K4me2 and H2A.Z have a hierarchical relationship; H3K4me2 is necessary for H2A.Z incorporation, but H3K4me2 is not dependent on H2A.Z incorporation (Light et al., 2010; Sump et al., 2022).

Memory leads to recruitment of the RNAPII pre-initiation complex (PIC) lacking Kin28 (Cdk7 in other systems), the kinase that phosphorylates serine 5 of the carboxy-terminal domain (CTD) of RNAPII. This form of PIC includes Ssn3 (Cdk8 in other systems), a kinase Mediator module (D’Urso et al., 2016). Thus, although RNAPII is recruited during memory, the ultimate phosphorylation of the CTD of RNAPII leading to initiation is blocked, leading to an inactive, poised state. This poised state requires Ssn3/Cdk8 kinase activity; inhibition of Ssn3 leads to rapid (but reversible) loss of RNAPII from the *INO1* promoter and slows the rate of reactivation of the gene and disrupts the competitive fitness advantage of memory (Sump et al., 2022). Thus, the association of poised RNAPII is essential for the output of memory.

### Inheritance of *INO1* epigenetic transcriptional memory

What is the molecular mechanism of inheritance of transcriptional memory? One obvious candidate is the post-translational modifications of chromatin associated with memory. During memory, nucleosomes at the *INO1* promoter and over the 5′ end of the gene exhibit H3K4me2 (D’Urso et al., 2016; Light et al., 2010). Methylation of H3K4 is associated with active transcription (Santos-Rosa et al., 2002) and is the result of a cascade of events. Initially, there is the highly specific mono-ubiquitination of H2B K123 (H2B K120 in mammals) as deposited by interactions of the E2 ubiquitin-conjugating enzyme, Rad6, and the E3 ubiquitin-ligase enzyme, Bre1 (Wood et al., 2003). This properly established mono-ubiquitination of H2BK123 is required for the Swd2(Cps35)-dependent recruitment of the Set1/COMPASS complex.

Methylation of H3K4 is also dependent upon the PAF1 complex, which consists of five, highly conserved subunits Paf1, Rtf1, Cdc73, Leo1, and Ctr9 (Krogan et al., 2002). A sixth subunit known as Ski8 is also found in the mammalian PAF1 complex (Zhu et al., 2005). Rad6/Bre1 physically interacts with the PAF complex, which interacts with active RNAPII, suggesting that active RNAPII stimulates H2BK123Ub and then H3K4 methylation (Conaway et al., 2002; Ng et al., 2003b; Kim and Buratowski, 2009). Rtf1, Ctr9, and Paf1 are essential for H3K4 methylation and Rtf1 is required for H2BK123 mono-ubiquitination (Ng et al., 2003a). Curiously, while Leo1 and Cdc73 are part of the PAF1 complex, these subunits are not required for H3K4 methylation globally (Krogan et al., 2003; Wood et al., 2003).

H3K4me2 associated with memory is distinct from that observed during active transcription in several important ways. First, it is RNAPII-independent, suggesting that it is deposited by a distinct mechanism. Second, unlike the H3K4 methylation associated with active transcription, the H3K4me2 observed during memory is dependent on Nup100, Set3, Leo1, and Sfl1/Hms2 (D’Urso et al., 2016; Light et al., 2013, 2010 Sump et al., 2022). Third, unlike H3K4 methylation associated with active transcription, which is rapidly lost upon repression (in the absence of memory (Sump et al., 2022)), H3K4me2 associated with memory can be epigenetically inherited for several generations. Specifically, although Sfl1 is required for H3K4me2 during memory, if Sfl1 is conditionally inactivated after memory has been established, H3K4me2 remains associated with the *INO1* promoter for 3–4 generations (Sump et al., 2022). Therefore, Sfl1 is required for the establishment, but not the inheritance, of H3K4me2 during *INO1* transcriptional memory. After establishing this chromatin state, Sfl1 is dispensable. However, the reader protein Set3 is continuously required; inactivation of Set3 leads to rapid loss of H3K4me2 during memory (Sump et al., 2022).

**FIGURE 2:**
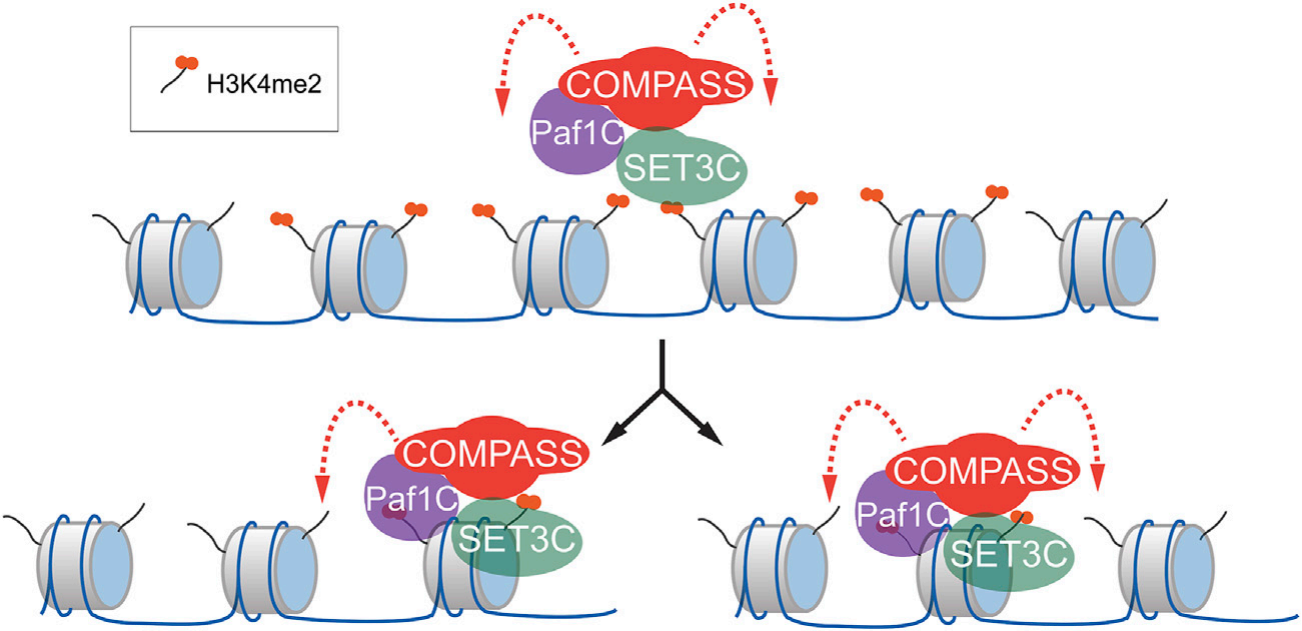
Model for the mechanism of inheritance of H3K4me2 during epigenetic transcriptional memory. During memory, H3K4me2 is both protected from demethylation by SET3C and, through interaction with COMPASS, SET3C H3K4me2 is maintained (top). Following DNA replication (bottom), H3K4me2-modified nucleosomes are reincorporated locally, along with unmodified nucleosomes. SET3C binding of H3K4me2 through its PHD domain facilitates recruitment of COMPASS/PAF1C, which demethylates unmodified nucleosomes nearby.

Inheritance of H3K4me2 during memory may reflect reader-writer crosstalk. Histone modifications such as H3K9me3 and H3K27me3 can be epigenetically inherited (Hansen et al., 2008; Methot et al., 2021). These marks are associated with very stable silencing and generally cover large regions of the genome (tens to hundreds of kilobases). The inheritance of such marks is thought to occur through 1) the local reincorporation of nucleosomes after DNA replication, 2) recognition of the modifications by reader proteins and 3) recruitment of writer proteins to reinforce these modifications (Reinberg and Vales, 2018; Escobar et al., 2019). H3K4me2 found near the *INO1* promoter is deposited by Spp1- COMPASS and is recognized by SET3C. These complexes physically interact, suggesting that they may constitute a reader-writer pair that re-establishes the H3K4me2 following DNA replication (Sump et al., 2022; Figure 2).

H3K9 and H3K27 are associated with very stable silencing that can persist for many generations, while *INO1* transcriptional memory is relatively short-lived. What accounts for this difference? First, while H3K9 and H3K27 can be inherited, these modifications are normally stimulated and reinforced by several cis-acting mechanisms, including transcription factors, siRNAs, Polycomb Response Elements, etc. Thus, cells do not rely on chromatin alone to determine the transcriptional regulation of these loci and these additional mechanisms likely contribute to their stability. Second, while most regions marked with H3K9me3 or H3K27me3 are large and represent hundreds of nucleosomes, the modifications associated with *INO1* memory are much more localized, encompassing a small number of nucleosomes. Given the same fidelity of recognizing and reintroducing histone modifications, the efficiency and fidelity of chromatin-dependent inheritance should scale with the number of nucleosomes. Thus, it is possible that, if the size of the region marked by the memory-specific chromatin signature were larger, the chromatin state would be inherited for many more generations.

## Summary

Recent studies expand both the knowledge of the molecular machinery to establish and maintain epigenetic transcriptional memory and our understanding of epigenetically inherited histone marks. Histone marks associated with repression can be inherited through reader-writer or reader-easer crosstalk in order to maintain stable transcriptional silencing (Reinberg and Vales, 2018). However, the finding that H3K4me2 associated with transcriptional memory is both RNAPII-independent and can be maintained through COMPASS-SET3C interactions, highlights the importance of heritable histone marks that stimulate transcription.
